# Progress of Surgery

**Published:** 1901-07-06

**Authors:** 


### PROGRESS OF SURGERY.

### THE SURGERY OF THE KIDNEY.

**Renal Calculus.**—Alban Doran[1] has recorded an instructive case of painless calculous pyonephrosis in which he successfully removed the kidney. He brought forward the case because surgeons were apt to exclude the diagnosis of calculous pyonephrosis when they found pain absent, even though the kidney was enlarged, on the ground that that disease must always be more or less painful. The case was also remarkable for the absence of fever, although there was much suppuration. The patient was a woman, 38 years of age. Her husband had been subject to stricture and cystitis. For a few months she had suffered from purulent vaginal discharge. For a year she had noticed a swelling in the right side, which ached when she put on her stays, but she was always comfortable when undressed. There was no history of rigors, fever, hæmaturia, or renal colic. The swelling was a large movable right kidney. It was not tender. The organ was removed through Langenbuch's incision with great ease. The ureter, which was reduced to a thin cord, was fixed in the lower end of the incision. The patient was in good health three months later. The kidney had become a cyst containing a pint of *pus laudabile.* The lowest calices were blocked by a small rough calculus and formed a bilocular cyst. The upper part of the kidney was almost a unilocular cyst, formed through complete stricture of the ureter at its origin from the pelvis. Probably the infection was gonococcal and therefore ascending. It is suggested in explanation of the complete stricture of the ureter, that the loose calculus may have formerly obstructed it and then become loose and that the obstruction had been completed through inflammation and contraction below the stone. A specimen from a similar case is preserved in the College Museum. It was removed by Meredith. Loose stones were found in the pelvis, and the ureter was completely strictured. Another specimen showed such stricture apparently produced independently of stone. Doran remarks that it was fortunate he removed the kidney while the fat and connective tissue around the kidney remained healthy. This enabled the pus-cyst to be removed easily and without rupture. The observation of Noble (Philadelphia) is quoted, who had said that "previous experience had shown that when the system was relieved of the pressure of one suppurating kidney, the other, even though not perfectly normal, did its work better." The ureter, being atrophied, did not require removal by Howard Kelly's very radical nephro-ureterectomy. Bruce Clarke's experience is quoted, viz., that painless pyonephrosis without stone is almost the rule, but that the frequency of painless calculous pyonephrosis is doubtful. Doran's case shows that a patient may go about with one kidney converted into a bag of pus and yet appear to be in excellent health.

**Treatment of Septic Infections of the Kidneys.**—L. L. McArthur,[2] of Chicago, has recently discussed this topic. He considered the subject under the headings of:—(1) Internal medication; (2) Local application, *e.g.*, ureteral laverage and antiseptics; (3) Surgical interference, either nephrotomy or nephrectomy. The nature of the infection should be determined by bacteriological examination, and the question of the involvement of one or both kidneys settled. Efforts should then be made to remove the source of infection, to destroy or inhibit the organism present, and to provide free exit for the products of infection. If the infection was from extension from cystitis, vigorous treatment of that must be instituted. The choice of medicaments was not of so much moment as the systematic carrying out of a rigid hygiene of the bladder. If the urine be acid, especially so when the gonococcus and bacillus coli communis were present, the urine should be rendered alkaline, and the elimination from the kidneys of much fluid insured. Antiseptics should be given. The kidneys should be assisted by lightening their work through good elimination from the bowels. The bacillus coli being the most frequent cause of renal infection, it would be well to give intestinal antiseptics. If after reasonable trial of these methods, and the use of ureteral lavage *à la* Caspar, Albarran, and Kelly, the case still progressed unfavourably, as the majority perhaps would, nephrostomy should be performed by the retro-peritoneal route. A muscle-splitting operation might be done. He did not think it possible or wise often to deliver the kidney, and felt sure he had seen a death from too great traction being made on the pedicle of the right kidney. This had constricted the inferior vena cava, and cut off the return flow to the heart.

[1] Brit. Med. Jour., March, 1901. [2] Med. Rec., January, 1901.

